# Post stroke aspiration pneumonia, associated factors, and treatment outcome among stroke patients admitted to Tibebe Ghion Specialized Hospital, Bahir Dar, Ethiopia

**DOI:** 10.3389/fstro.2024.1410657

**Published:** 2024-11-08

**Authors:** Molla Dessalegn Nigus, Ergoye Melese Sendek, Mulugeta Biyadgie Ewunetu, Abel Belete Cherkos, Awol Arega Yimer

**Affiliations:** ^1^College of Medicine and Health Sciences, School of Medicine, Bahir Dar University, Bahir Dar, Ethiopia; ^2^Department of Epidemiology and Biostatistics, College of Medicine and Health Sciences, Bahir Dar University, Bahir Dar, Ethiopia; ^3^Department of Midwifery, College of Medicine and Health Sciences, Arba Minch University, Arba Minch, Ethiopia

**Keywords:** post-stroke aspiration pneumonia, prevalence, stroke, Tibebe Ghion Specialized Hospital, treatment outcome

## Abstract

**Background:**

Post-stroke aspiration pneumonia is a serious lung infection that occurs when stroke patients inhale food, saliva, liquid, vomit, or foreign objects. It is the leading cause of death for stroke patients worldwide, which accounts for 60% of stroke-associated deaths. Little is known about its prevalence, adverse outcomes, and determinants in Ethiopia. Therefore, this study aims to evaluate the prevalence of post-stroke aspiration pneumonia and associated factors in stroke patients admitted to the Tibebe Ghion Specialized Hospital.

**Methods and materials:**

A retrospective cross-sectional study was conducted on 242 stroke patients admitted to the neurology unit of a medical ward from January 1, 2019, to December 30, 2020. Data were collected from the patients' cards using a pre-developed data collection tool. The collected data was coded, cleaned, and entered into Epi-Info version 7.25, and then exported to SPSS 26 for further analysis. Logistic regression analyses were performed to identify factors associated with aspiration pneumonia. The adjusted odds ratio with the corresponding 95% confidence interval and a *p* < 0.05 were noted to declare variables had a significant association.

**Results:**

The mean age of the patient was 61.15 ± 13.959 years with a minimum age of 21 and maximum age of 102 years. Males account for 55.8% and females for 44.2%. Out of 242 patients post-stroke aspiration pneumonia was identified in 23.1% of patients, and 55.4% of stroke patients improved, while 23.2% died from post-stroke aspiration pneumonia. The study found that having dysphagia (AOR = 3.05; 95% CI: 1.13, 8.21), feeding through a Nasogastric tube (AOR = 10.58; 95% CI: 4.58, 24.42), and a GCS level of 8–12 (AOR = 2.58; 95% CI: 1.04, 6.42) were independent predictors of post-stroke aspiration pneumonia.

**Conclusion:**

This study found a high prevalence of post-stroke aspiration pneumonia and its associated mortality. Stroke patients with dysphagia, low levels of consciousness, and those who are fed through a tube are at a higher risk of developing this condition. Therefore, it is crucial to provide special care to stroke patients with these conditions. The study also highlights the importance of assessing the practice of preventative measures for PSAP among stroke patients in this study setting.

## Introduction

Patients who have had a stroke are at a higher risk of aspirating gastric contents due to several factors. For example, stroke patients with neurological damage are more likely to experience aspiration due to weakened swallowing reflexes. Additionally, patients who require mechanical ventilation through an endotracheal tube are at an increased risk of aspiration, as this can impede typical aspiration defense mechanisms such as coughing. This elevated risk of aspiration can lead to Post-stroke Aspiration Pneumonia (PSAP) (Grossmann et al., 2021). After a stroke, about one-third of hospitalized stroke patients experience PSAP which is one of the most common medical complications associated with worse clinical outcomes (Armstrong and Mosher, 2011). PSAP is an infection of the lower respiratory tract that results from the entrance of gastrointestinal contents into the lungs (Finlayson et al., 2011).

PSAP is the burden of all countries across the world with a global pooled prevalence of 14.03% (Chen et al., 2021). However, the prevalence of PSAP varies significantly among different economic regions. In high-income countries (HICs), the magnitude of PSAP ranges from 37 to 78% in Germany (Cieplik et al., 2020), 9 to 10% in Switzerland (Seystahl et al., 2023), 2.3 to 44% in China (Liu et al., 2022), 60.8% in the United Kingdom (UK) (Teh et al., 2018), and 5.1% in the United States of America (USA) (Chang et al., 2013). The magnitude of PSAP also varies across low- and middle-income countries (LMICs). For instance, 15.56% in Pakistan (Khalid et al., 2022), 46% in Egypt (Belal et al., 2020), 17.3% in Nigeria (Sadiq et al., 2023), and 26 % in Zambia (Prust et al., 2021). In Ethiopia, the prevalence of PSAP ranges from 19.8% to 39.4% (Fekadu et al., 2019; Assefa et al., 2022; Lidetu et al., 2023; Mamushet et al., 2015; Asgedom et al., 2020). PSAP is the most substantial contributor to long-term morbidity and it has the highest attributable death rate among all post-stroke medical complications (Armstrong and Mosher, 2011; Heuschmann et al., 2004). About 60% of stroke patients die from PSAP (Prust et al., 2021).

In many stroke patients, PSAP may progress into systemic infection, sepsis, and then acute respiratory failure secondary to Aspiration-related acute respiratory distress syndrome (ARDS) frequently occurs which leads to prolonged hospitalization and poor treatment outcomes (Arnold et al., 2016; Rohweder et al., 2015; Zhao et al., 2015). More than 30% of patients with a hemorrhagic stroke and 6% of patients with ischemic stroke were intubated for respiratory failure secondary to PSAP (Gujjar et al., 1998).

Both the sociodemographic and clinical conditions of the patient were linked with PSAP. Among sociodemographic variables; sex, advanced age, and residence contribute to the occurrence of Aspiration pneumonia in stroke patients (Cieplik et al., 2020; Lidetu et al., 2023; Li et al., 2019). Furthermore, stroke patients who had dysphagia, severe stroke, nasogastric tubes, Lower Glasgow Coma Scale (GCS), dysphagia, feeding modalities, facial palsy, vomiting, other medical comorbidities like diabetes, coronary heart disease, myocardial infarction, atrial fibrillation, heart failure, and patients who didn't receive prophylaxis antibiotics were more likely to develop PSAP (Lidetu et al., 2023; Oliveira et al., 2015; Assefa et al., 2022; Fekadu et al., 2019; Mamushet et al., 2015; Asgedom et al., 2020). Furthermore, underscoring the process of care-related factors is crucial, as these factors are important predictors of PSAP and other stroke-associated complications (Soares et al., 2022).

To decrease the incidence of PSAP different studies recommended patient mobilization, appropriate head and neck positioning, early provision of prophylaxis antibiotics (within 24 hours), anti-emetics, and Beta-blockers for post-stroke patients (Grossmann et al., 2021; Rashid et al., 2020). In addition for stroke patients with dysphagia Nasogastric Tube (NGT) feeding and early diagnosis and management of dysphagia, intubating those patients with low GCS, and oral hygiene maintenance, play vital roles in the prevention of PSAP (Grossmann et al., 2021).

Identifying factors that determine the likelihood of developing PSAP is crucial to establishing preventative measures, delivering prompt treatment, and lowering the morbidity and mortality associated with PSAP. There is a paucity of data regarding factors associated with PSAP, despite the fact that it has been repeatedly reported as the first leading cause of morbidity and mortality in stroke patients in the past years in Ethiopia. Therefore, the main aim of this study is to assess the magnitude of PSAP and associated factors in stroke patients in Tibebe Ghion Specialized Hospital (TGSH).

## Methods and material

### Study area and period

The study was conducted at TGSH in Bahir Dar City, Ethiopia from May 1 to June 30, 2021. TGSH is a tertiary university teaching hospital with a 450-bed capacity. The hospital receives patients referred from across the Amhara region. Since the end of 2018, the hospital has had a dedicated stroke unit within the Neurology ward. This unit consists of separate male and female sections, equipped with 18 beds, suction, monitors, and imaging modalities (CT and MRI). On average, the unit provides care to 130 stroke patients annually. The services offered by the Stroke Unit include emergency response to recognize stroke symptoms for immediate medical interventions, acute care (excluding surgical interventions), monitoring, rehabilitation, education and support, secondary prevention, and follow-up care.

### Study design

A facility-based retrospective cross-sectional study design was employed.

### Populations

#### Study populations

Patient records of stroke patients admitted to the neurology ward of Tibebe Ghion Specialized Hospital from 01 January 2019 to 30 December 2020 and fulfilled the inclusion criteria were the study population.

### Selection criteria

Charts of stroke patients who were admitted to the neurology ward and had a complete medical record were included in the study. Whereas, patient charts with query diagnosis of stroke were excluded from the study.

### Sample size determination and sampling technique

The sample size was determined using the single population proportion formula on open epi stat calc by considering the following assumptions: 95% level of confidence, 5% margin of error as well as 19.8% of stroke patients estimated to develop PSAP in a study done in Jimma University Hospital (Fekadu et al., 2019).


$$\begin{array}{l} n=\frac{{\left({\text{Z}}_{\text{α}/2}\right)}^{2}*\text{P}\left(1-P\right)}{d^{2}}=\frac{{\left(1.96\right)}^{2}*0.198\left(0.802\right)}{{\left(0.05\right)}^{2}}=\text{244},\;Where \end{array}$$

n = the required sample size

Z_α/2_ = the value of the standard normal curve at 95% confidence level = 1.96

p = proportion of PSAP = 19.8%

d = the degree of precision is 5%

After adding a 5 % non-response rate for incomplete cards the final sample size becomes 256. There was a total of 260 stroke patients were admitted to The Hospital from January 2019 to December 2020, all of whom were included as study participants due to the manageable population size.

### Study variables

#### Dependent variable

Post-stroke Aspiration pneumonia (1 = yes and 0 = no).

#### Independent variable

**Sociodemographic variables:** Age, Sex, Marital status, Occupation, Residence, and level of education.

**Clinical variables:** Type of stroke, level of consciousness, type of feeding, neurologic deficit, mechanical ventilation, dysphagia, medical unit at first admission, time of arrival in hours, length of hospital stay in days, other medical comorbidities, and patient outcome.

### Operational definitions

**Aspiration pneumonia:** is an infection of the lungs that results from the entry of stomach or throat fluid, which may contain bacteria, mineral oil, water, salt, ingested liquids, or food particles, into the lower airways (Michael Klompas, 2023).

**Post-stroke Aspiration Pneumonia:** is aspiration pneumonia in patients admitted with stroke, which is diagnosed when at least two of the following clinical variables are present: temperature higher > 38°C, oxygen saturation <92%, respiratory rate > 20 breath/minute, cough, rhonchi, witnessed aspiration event, and antibiotic initiated for clinically suspected aspiration pneumonia with white blood cell count > 10,000 x 10^9^/L on complete blood count and infiltrate on chest X-ray (Prust et al., 2021). For this study, the diagnosis of PSAP was taken from the patient chart that was documented by a senior physician.

**Dysphagia** is a condition in which swallowing is difficult or impaired, and it is diagnosed through a bedside screening test such as the Gugging Swallowing test (Yoshimatsu et al., 2022). For this study, we obtained the diagnosis from the patient's chart.

### Data collection tool and procedure

The data was collected using a structured and standardized data extraction checklist, which was developed after reviewing similar studies conducted in various parts of the world. The tool was developed in English and pre-tested for validity before the data collection format was finalized. Before the actual data collection, 3 Year II internal medicine residents were recruited as data collectors and 1 Year III internal medicine resident student was selected as a supervisor. They were trained for 2 days on the purpose of the research, the questions included in the data extraction checklist, as well as the process of completing the checklist. The medical registration numbers (MRN) of all patients who were admitted to the neurology ward between January 1st, 2019, and December 30, 2020, were obtained from the patient registration book. The data collectors then retrieved each patient's card from Tibebe Gion Specialized Hospital's patient card store and filled out the data extraction sheet based on the information obtained.

### Data quality control

In order to ensure the quality of the data, a pretest of the checklist was conducted one week prior to the data collection period on 5% of the total sample size. The collected data was thoroughly reviewed and checked for completeness before being entered; any incomplete data was discarded. Data collectors and the supervisor received 1 day of training. During the data collection period, both the supervisor and principal investigator provided adequate supervision. The collected checklists were spot-checked daily. Finally, the collected data underwent a thorough check for completeness, accuracy, and clarity by the principal investigator and the supervisor.

### Data processing and analysis

The collected data were entered into EPI-data manager version 7.25 software after being checked for completeness manually and exported to SPSS version 26. On SPSS The data was analyzed through descriptive analyses to determine the frequency and percentages. A bivariate logistic regression analysis was then conducted on each independent variable to identify any factors that could contribute to the outcome of interest (PSAP). Variables with a *p* < 0.25 were selected for the multivariable logistic regression analysis. Each parameter was estimated to identify whether it was significant, and a *P* < 0.05 was used as a cut point. The crude and adjusted odds ratios, together with their corresponding 95% confidence intervals, were then calculated and interpreted accordingly. Finally, the variance inflation factor (VIF) was used to assess the multicollinearity assumptions, and the Hosmer and Lemeshow test was conducted to check the goodness of fit for the final model.

### Ethical consideration

The Institutional Review Board (IRB) of Bahir Dar University, College of Medicine and Health Sciences reviewed and approved the study protocol, and ethical clearance was granted. In addition, a permission letter was obtained from Tibebe Gion Specialized Hospital.

## Results

### Sociodemographic characteristics of the patients

Out of 260 charts, 18 were incomplete and discarded resulting in a response rate of 93.1%. The mean age of the patients in the study was 61.15 ± 13.959 years, with the youngest patient being 21 years old and the oldest being 102 years old. Of the patients, 55.8% were male and 44.2% were female, with a male-to-female ratio of 1.26. About one-third of the study group had attained a college education (31.4%), while 26.9% were had no formal education. The most common occupation was farming (32.6%). The majority of patients were from rural areas (57.9%), while 42.1% were from urban areas (Table 1).

**Table 1:** Socio-demographic characteristics of stroke patients admitted to the TGSH stroke unit between Jan 2019 and Dec 2020.

**Variables**	**Category**	**(*N* = 242)**	**Number of patients with PSAP *N* = 56**	**Number of patients Without PSAP; *N* = 186**
Age in years	<65 years	141(58.3%)	28	113
	65 and above	101(41.7%)	28	73
Sex	Male	135(55.8%)	31	104
	Female	107(44.2%)	25	82
Marital status	Single	7(2.9%)	0	7
	Married	218(90.1%)	50	168
	Divorced	9(3.7%)	2	7
	Widowed	8(3.3%)	4	4
Religion	Orthodox	172(71.1%)	45	127
	Muslim	45(18.6%)	6	39
	Protestant	25(10.3%)	5	20
Level of education	No formal education	65(26.9%)	17	48
	Elementary	51(21.1%)	14	37
	High school	50(20.7%)	7	43
	College and above	76(31.4%)	18	58
Occupation	Farmer	79(32.6%)	19	60
	Housewife	49(19.8%)	8	40
	Government employee	54(22.3%)	11	43
	NGO^*^	12(5%)	5	7
	Retired	26(10.7%)	8	18
	Jobless	15(6.2%)	4	11
	Other^**^	8(3.3%)	1	7
Place of residency	Urban	102(42.1%)	22	80
	Rural	140(57.9%)	34	106

NGO^*^, non-governmental organization. Other^**^: merchant = 5, student = 1, priest = 2.

### Clinical characteristics of stroke patients

Out of a total of 242 stroke patients, 177 stroke (73.1%) had hypertension, 57 (23.6%) had atrial fibrillation, and 20 (8.3%) were diabetic. The majority of the patients, 144 (59.5%), arrived at the hospital more than 12 h after experiencing stroke symptoms. In terms of feeding, 71 (29.3%) of stroke patients were fed via nasogastric tube. Regarding the level of consciousness, 152 (62.8%) patients scored 14 or 15 on the Glasgow Coma Scale, while only 19 (7.9%) scored 8 or below. Additionally, 222 (91.7%) had facial palsy, and 46 (19%) had dysphagia as focal neurologic deficits (Table 2).

**Table 2:** Clinical characteristics of stroke patients admitted to the TGSH stroke unit between Jan 2019 and Dec 2020.

**Variables**	**Frequency (n)**	**Percentage (%)**
**Preexisted medical condition**		
Hypertension	177	73.1%
Diabetics	20	8.3%
Atrial fibrillation	57	23.6%
Rheumatic heart disease	21	8.7%
Others^**^	6	2.46%
**Mechanical ventilation**		
Yes	9	3.7
No	233	96.3
**Time of arrival**		
Within 4.5 h	20	8.3
4.5–12 h	78	32.2
Above 12 h	144	59.5
**Level of consciousness**		
≤ 8	19	7.9%
9–13	71	29.3%
14–15	152	62.8%
**Feeding type**		
Oral feeding	171	70.7
Nasogastric tube feeding	71	29.3
**Initial admission**		
Neurology ward	235	97.1
Intensive care unit	7	2.9
**Type focal neurologic deficit**		
Facial palsy	222	91.7%
Dysphagia	46	19%
Speech deficit	144	59.5%
**Stroke type**		
Hemorrhagic	96	39.7%
Ischemic	146	60.3%

Others^**^1 = takayasu arthritis, 1 = asthma, 2 = DVHD, 1 = DCMP, 1 = Seizure.

### Prevalence of PSAP and treatment outcome of stroke patients

**Figure 1:**
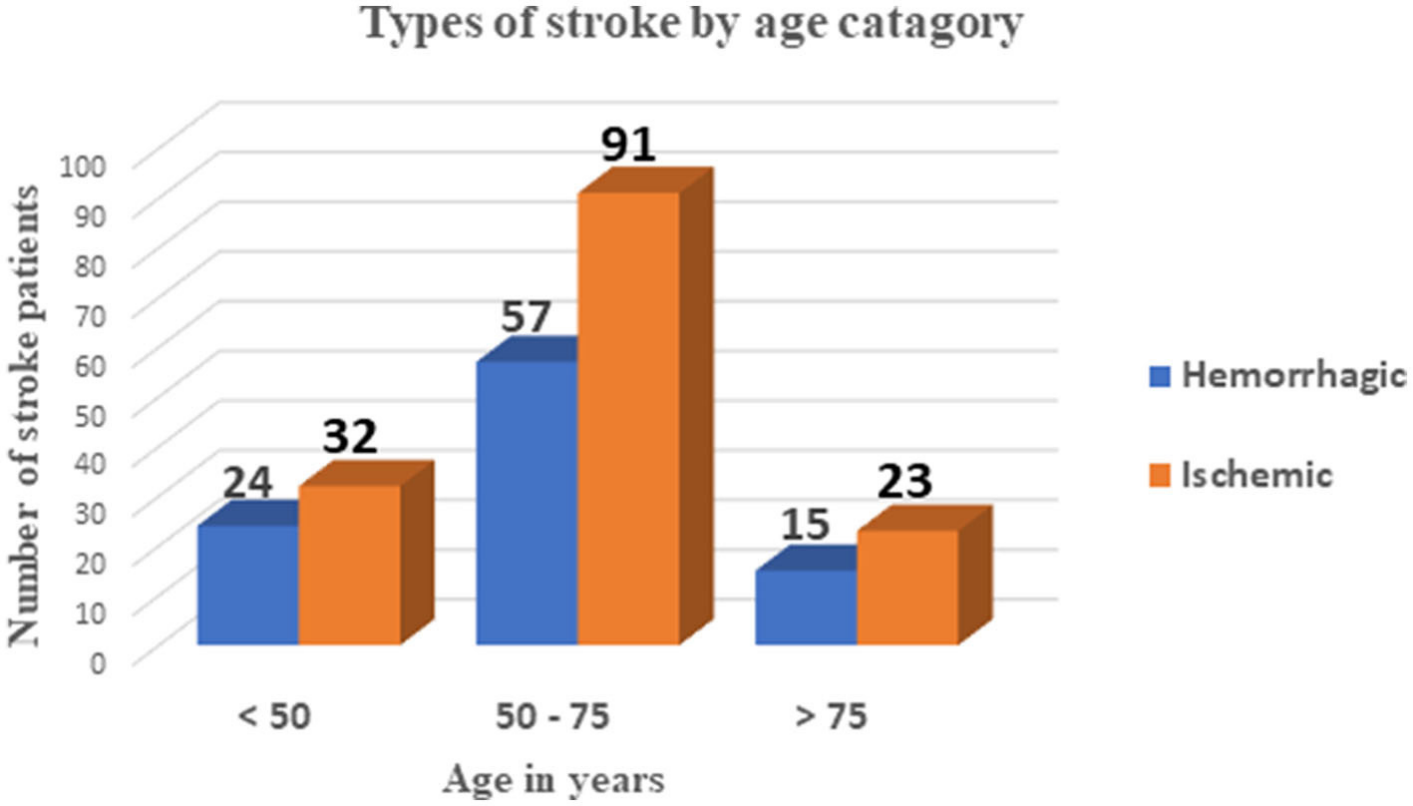
Types of strokes by age category among stroke patients admitted to the TGSH stroke unit between Jan 2019 and Dec 2020.

**Figure 2:**
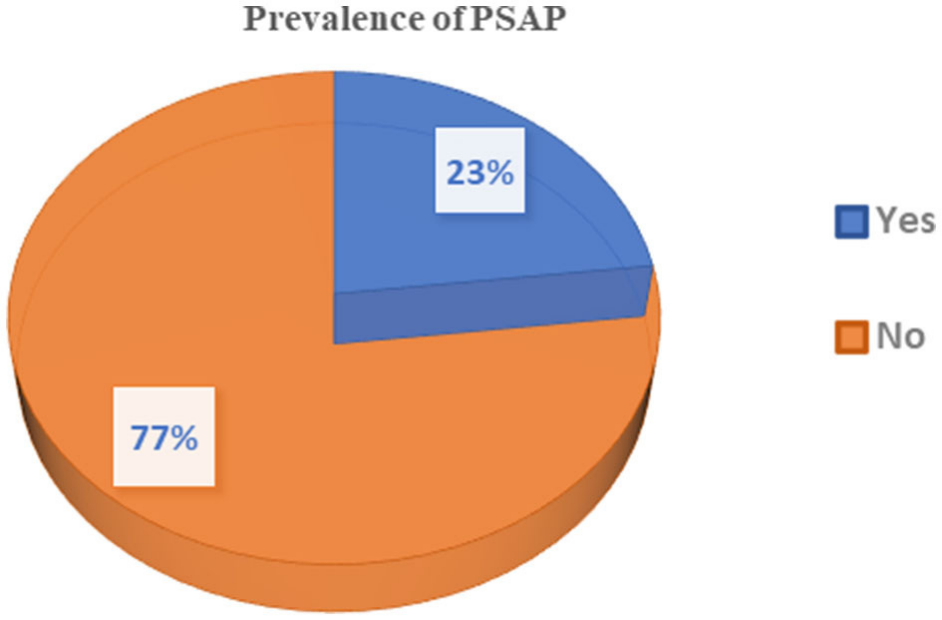
Prevalence of PSAP among stroke patients admitted to the TGSH stroke unit between Jan 2019 and Dec 2020.

Among these stroke patients, 188 (77.7%) showed improvement and were discharged after treatment. Of the 56 patients with PSAP, 13 (23.2%) died due to respiratory failure, while only one patient died out of the 186 stroke patients without PSAP, and that was due to cardiac failure (Table 3). As shown in Figure 1, most stroke patients in this study had an ischemic type of stroke, and their ages ranged from 50 to 70 years. Out of 242 stroke patients, 56 (23.1%) developed post-stroke aspiration pneumonia (PSAP) (Figure 2).

**Table 3:** Outcome of stroke patients admitted to the TGSH stroke unit between Jan 2019 and Dec 2020.

**Outcome**	**Patients with PSAP (frequency and percentage)**	**Patients without PSAP (frequency and percentage)**	**Total (frequency and percentage)**
Improved and discharged	31 (55.4%)	157 (84.4%)	188 (77.7%)
Death	13 (23.2 %)	1 (0.5%)	14 (5.8%)
Went to home against medical advice	11 (19.6%)	20 (10.8%)	31 (12.8%)
Referred	1 (1.8 %)	8 (4.3%)	9 (3.7%)
Total	56 (100%)	186 (100%)	242 (100%)

### Factors associated with post stroke aspiration pneumonia

In the bivariate logistic regression analysis, variables like time of arrival to the hospital, age, educational level, speech deficit, level of consciousness in GCS, dysphagia, duration of hospital stay, feeding type, and atrial fibrillation were associated with PSAP at a *p* < 0.25. These factors, along with factors that were significantly associated in another study such as sex of patients, and residence, were included in the final model for multivariable logistic regression analysis. The results showed that the level of consciousness, dysphagia, and type of feeding were significantly associated with PSAP at a *p* < 0.05 with a 95% confidence interval.

This study found that the prevalence of PSAP was 3.05 times higher (AOR = 3.05; 95% CI: 1.13, 8.21) in stroke patients with dysphagia, compared to stroke patients without dysphagia. Additionally, the odds of developing PSAP were 10.58 times higher (AOR = 10.58; 95% CI: 4.58, 24.42) in patients who were fed via Nasogastric tube (NGT) than in those who were fed orally (PO). The likelihood of developing PSAP was also 2.58 times higher (AOR = 2.58; 95% CI: 1.04, 6.42) in patients with a Glasgow Coma Scale (GCS) level of 8–12, and 2.01 times higher (AOR = 2.01; 95% CI: 0.45, 9.09) in patients with a GCS level below 8, compared to patients with a GCS level of 14 and 15 (Table 4).

**Table 4:** Logistic regression analysis result of factors associated with PSAP among stroke patients admitted to the TGSH stroke unit between Jan 2019 and Dec 2020.

**Variables**	**PSAP**		**Odd Ratio (95% CI)**		***p*-value for AOR**
	**Yes**	**No**	**Crude odds ratio**	**Adjusted odds ratio (AOR)**	
**Age in completed years**					
<65	28	113	1	1	
65 and above	28	73	1.55 (0.85, 2.82)	0.86 (0.37, 1.97)	0.716
**Sex**					
Male	31	104	0.98 (0.54, 1.78)	1.52(0.65, 3.54)	0.329
Female	25	82	1	1	
**Educational level**					
No formal education	17	48	1.14 (0.53, 2.45)	1.04(0.27, 3.97)	0.951
Elementary	14	37	1.22(0.54, 2.74)	1.04 (0.28, 3.91)	0.952
High school	7	43	0.53 (0.20, 1.37)	0.45 (0.12, 1.74)	0.248
College and above	18	58	1	1	
**Residence**					
Urban	22	80	0.86 (0.47, 1.58)	0.72 (0.23, 2.24)	0.571
Rural	34	106	1	1	
**Time of arrival**					
Within 4.5 h	7	13	1	1	
4.5–12 h	12	66	0.34 (0.11, 1.02)	1.44(0.32, 6.59)	0.637
Above 12 h	37	107	0.64 (0.24, 1.73)	0.48 (0.18, 1.29)	0.144
**Dysphagia**					
Yes	26	20	7.19 (3.57,14.50)^*^	**3.05(1.13, 8.21)**	**0.027**
No	30	166	1	1	
**Speech deficit**					
Yes	43	101	2.78(1.40, 5.52)^*^	1.28(0.54, 3.07)	0.577
No	13	85	1	1	
**Level of consciousness in GCS**					
<8	11	8	8.58 (3.09, 23.80)^*^	2.01 (0.45, 9.09)	0.364
9–13	24	47	3.19 (1.62, 6.25)^*^	**2.58 (1.04, 6.42)**	**0.042**
14–15	21	131	1	1	
**Atrial fibrillation**					
Yes	17	40	1.59 (0.82, 3.10)	1.29 (0.51, 3.26)	0.594
No	39	146	1	1	
**Types of feeding**					
NGT	43	28	18.67 (8.91,39.09)^*^	10.58 (4.58, 24.42)	**<** **0.001**
PO	13	158	1	1	
**Duration of hospital stay**					
1**–**7 days	30	108	1		
7**–**14 day	20	70	1.03 (0.54, 1.95)	0.94 (0.39, 2.26)	0.887
above 14 days	6	8	2.70 (0.87, 8.39)	2.97 (0.62, 14.12)	0.172

^*^Indicates variables had a significant association at p <0.05 in bivariate logistic regression analysis, and 1 = the reference category in the model. Bolded values in the table indicate variables with significant associations with PSAP at *p* < 0.05 in the logistic regression analysis.

## Discussion

According to this study, the prevalence of PSAP was found to be 23.1% with a 95% CI of (18%, 28%). This finding is consistent with the results from studies conducted in Zambia (26%) (Prust et al., 2021), as well as in teaching hospitals in Ethiopia, such as Jimma University Medical Center (19.8%) (Fekadu et al., 2019), and Felege Hiwot Referral Hospital (23.06%) (Lidetu et al., 2023). However, it is lower than the prevalence found in other studies, such as those conducted in Black Lion Specialized Hospital (33.8%) (Mamushet et al., 2015), Ayder Comprehensive Specialized Hospital (39.4%) (Asgedom et al., 2020), China (44%) (Liu et al., 2022), and Egypt (46%) (Belal et al., 2020). Conversely, it is higher than the prevalence found in studies conducted in Nigeria (17.3%) (Sadiq et al., 2023), Pakistan (15.56%) (Khalid et al., 2022), Germany (8%) (Cieplik et al., 2020) and USA (5.1%) (Chang et al., 2013). The difference in the magnitude of PSAP could stem from differing diagnostic criteria worldwide. Different institutions use varying diagnostic criteria (Yoshimatsu et al., 2022) which may result in different magnitudes. Furthermore, the difference in care for post-stroke patients from institution to institution may also explain the discrepancies, as the process of care-related factors are important predictors of PSAP (Soares et al., 2022). For instance, some institutions provide prophylactic antibiotics for all stroke patients, while others do not. In Germany, Only 5 % of stroke units use prophylaxis antibiotics for post-stroke patients (Harms et al., 2012). Consensus among different stroke and stroke-related societies on uniform diagnostic criteria may enable us to know the exact figures of PSAP in different nations.

According to the findings of this study, 55.4% of stroke patients who underwent PSAP treatment showed improvement and were discharged, while 23.2% unfortunately passed away despite receiving treatment. It is worth noting that the PSAP-related mortality rate in this study is lower than that reported in Zambia (Prust et al., 2021), where it is 60%. This difference could be attributed to the fact that approximately 20% of patients with PSAP in this study left the hospital before completing their treatment against medical advice.

It has been consistently identified in various studies worldwide that dysphagia is a significant predictor of PSAP. Studies conducted in Germany (Cieplik et al., 2020), Bahir Dar (Lidetu et al., 2023), Iraq (Ali et al., 2020), South Korea (Chang et al., 2022), Nigeria (Watila et al., 2013), and Gondar (Assefa et al., 2022) all showed this. This study also found that the likelihood of PSAP is three times higher in stroke patients with dysphagia than in those without it. This is due to the loss of central nervous system control over the neural swallowing network in stroke patients with dysphagia, which results in a lack of coordination and dysfunction of the pharyngeal muscles (Pitts, 2014; Panebianco et al., 2020). This leads to impairment in the pharyngeal swallowing process and airway protective mechanisms, making patients more vulnerable to aspiration and thus more prone to PSAP (Pitts, 2014). Additionally, dysphagia following stroke can lead to malnutrition, which weakens immunity and increases the risk of pneumonia even with small amounts of aspiration (Feng et al., 2019). Using a bedside swallowing screening test for all stroke patients upon arrival and early diagnosis and management of dysphagia is crucial in reducing the incidence of PSAP.

According to this study, there is a significant association between the level of consciousness and PSAP. Specifically, stroke patients with a GCS level of 8–12 had greater odds of having PSAP, as compared to patients with a GCS level of 14 and 15. This is consistent with a study conducted in Nigeria (Watila et al., 2013), Germany (Dziewas et al., 2004), and Felege Hiwot Referral Hospital in Bahir Dar city (Lidetu et al., 2023). Patients who have had a stroke and have a low GCS level experience weakness or loss of their cough reflex, which is crucial for protecting against aspiration. Without this protective mechanism, patients are more prone to aspiration, which can lead to PSAP (Grossmann et al., 2021). In addition, in Ethiopia, there is a strong cultural practice of giving oral sips of water, milk, coffee, and tea to unconscious patients. However, this may augment the risk of aspiration and developing PSAP. Therefore, it is better to admit stroke patients with low GCS levels to the Intensive Care Unit (ICU) and take possible preventive measures to minimize the incidence of PSAP. Furthermore, strong health education programs are needed in the Ethiopian community to prevent this harmful practice.

Based on our study, NGT feeding is a strong factor that determines the likelihood of developing PSAP. The odds of developing PSAP were found to be 10.58 times higher in stroke patients who were fed via NGT than in those who were fed orally. This conclusion is supported by studies conducted in Germany (Cieplik et al., 2020), Egypt (Hamdy et al., 2023), Australian Hospitals (Brogan et al., 2014), Singapore (Mamun and Lim, 2005), and the Netherlands (Teramoto et al., 2006). The reason for this is that the NGT disrupts the usual defense barriers mechanically and it may allow a small amount of gastric contents to bypass the oropharynx, which can then be easily aspirated to the lungs in stroke patients. Additionally, the insertion of NGT may promote the colonization of microorganisms in the oropharynx (Brogan et al., 2014). Therefore, precautionary measures should be implemented during NGT insertion. Furthermore, patients with NGT had more severe strokes and impaired consciousness, which further increased the risk of PSAP (Prust et al., 2021; Eltringham et al., 2020). However, it's important to note that this study is cross-sectional and does not assess the timing of NGT insertion. Most likely, most of the patients may had NGT after aspiration, so the exact timing should be studied.

## Conclusion and recommendations

In this study, the prevalence of PSAP and PSAP-associated mortality was found to be high, at 23.1% and 23.2%, respectively. The three independent factors that were linked to PSAP in stroke patients were feeding type, dysphagia, and consciousness level. Therefore, stroke patients who have dysphagia, a lower GCS score, and who feed via NGT require special attention. Early detection and evaluation of dysphagia, the need for NGT feeding, and determining GCS levels are essential for stroke patients. Prompt, timely, and appropriate preventive measures should be taken to prevent aspiration, reduce the burden of PSAP, and improve treatment outcomes for patients with PSAP. For instance, the provision of anti-emetic drugs, ABC stabilization, swallowing training, and good positioning of stroke patients with dysphagia and low GCS score is optimal in preventing PSAP. Additionally, the practice of PSAP preventative measures among stroke patients needs to be assessed in this study setting.

## Limitations of the study

The finding of this study cannot be generalized to all stroke patients in Ethiopia as the study design is an institution-based cross-sectional study. Since this study was based on a retrospective chart review, multiple preventive measures of aspiration and process of care-related variables were not included in the study due to the lack of documentation in the charts. These variables should be investigated using an observational study design. Additionally, important predictors such as the patient's immune status, stroke severity (NIH Stroke Scale), modified Rankin Scale (mRS), duration of stay in the emergency unit, and timing of the NGT insertion were not included in this study due to the lack of documentation in the patient charts.

## Data Availability

The original contributions presented in the study are included in the article/supplementary material, further inquiries can be directed to the corresponding author/s.
